# Weak Ties That Bind: ICT Use, Social Relations, and Depressive Symptoms Among Older Adults

**DOI:** 10.1093/geroni/igab046.479

**Published:** 2021-12-17

**Authors:** Jess Francis, Noah Webster, Nour Fakhoury

**Affiliations:** University of Michigan, Ann Arbor, Michigan, United States

## Abstract

Information and communication technology (ICT) use has been associated with well-being among older adults. This link is often attributed to the fact that technology use facilitates connecting with social relations generally. What is less known, however, is the extent to which distinct dimensions of social relations uniquely influence how ICT use affects health. Thus, the importance of weak ties is receiving increased attention. Using data from the Detroit-based Social Relations Study collected in 2015, we examine the extent to which separate dimensions of weak ties (contact frequency and network size) mediate and moderate the link between technology use and depressive symptoms among adults age 65+ (n=213). A greater number of less close relations mediated the link as it was associated with technology use and fewer depressive symptoms. A moderating effect was also found as technology use was associated with fewer depressive symptoms only among those with lower contact frequency.

